# Across Bacterial Phyla, Distantly-Related Genomes with Similar Genomic GC Content Have Similar Patterns of Amino Acid Usage

**DOI:** 10.1371/journal.pone.0017677

**Published:** 2011-03-10

**Authors:** John Lightfield, Noah R. Fram, Bert Ely

**Affiliations:** Department of Biological Sciences, University of South Carolina, Columbia, South Carolina, United States of America; National Institutes of Health, United States of America

## Abstract

The GC content of bacterial genomes ranges from 16% to 75% and wide ranges of genomic GC content are observed within many bacterial phyla, including both Gram negative and Gram positive phyla. Thus, divergent genomic GC content has evolved repeatedly in widely separated bacterial taxa. Since genomic GC content influences codon usage, we examined codon usage patterns and predicted protein amino acid content as a function of genomic GC content within eight different phyla or classes of bacteria. We found that similar patterns of codon usage and protein amino acid content have evolved independently in all eight groups of bacteria. For example, in each group, use of amino acids encoded by GC-rich codons increased by approximately 1% for each 10% increase in genomic GC content, while the use of amino acids encoded by AT-rich codons decreased by a similar amount. This consistency within every phylum and class studied led us to conclude that GC content appears to be the primary determinant of the codon and amino acid usage patterns observed in bacterial genomes. These results also indicate that selection for translational efficiency of highly expressed genes is constrained by the genomic parameters associated with the GC content of the host genome.

## Introduction

Previous reports indicated that bacterial genomic GC content ranges from 25 to 75% [1], [2], [3]. Based on this observation, Sueoka [3],[4] predicted that differences in GC content would affect protein amino acid sequence even though the genetic code had not been elucidated at the time. Once DNA sequence technology was developed, Muto and Osawa [5] and later Knight et al. [6] analyzed the available nucleotide sequences from the genes of the available species of bacteria and showed that the GC content at all three codon positions increased with increasing genomic GC content. Similar results were obtained when 23 complete cyanobacterial genomes were analyzed [7]. In all three datasets, the biggest change in GC content was observed in the third codon position where most nucleotide changes do not change the amino acid sequence of the encoded proteins due to the redundancy of the genetic code. However, significant increases in GC content were observed in the first and second codon positions as well. Since nucleotide changes in the first and second codon positions usually result in changes in the amino acid sequence, these results suggested that genomic GC content has a significant impact protein amino acid sequence.

A more in depth analysis [8] showed that the use of amino acids encoded by GC-rich codons increased with increasing genomic GC content while the use of amino acids coded by GC-poor codons decreased with increasing GC content. Subsequently, Gu et al. [9] examined 15 individual proteins in 15 bacteria with a range of genomic GC content and showed that the amino acid composition of each protein was affected by changes in genomic GC content. A year later, Wilquet and Van de Casteele [10] performed an analysis of 22 protein families from 30 species of bacteria and archaea and concluded that the first codon position dominated the relationship between GC content and amino acid composition. Similar results were obtained when complete bacterial genomes were analyzed [11], [12]. Thus, bacterial codon usage patterns appear to reflect genomic GC content rather than phylogenetic relationships. This conclusion was reinforced by a subsequent study of 100 bacterial and archaeal genomes which showed that codon usage could be predicted by an analysis of the non-coding regions of the genomes [13].

Since an inspection of the completely sequenced bacterial genomes available in GenBank June 2010) suggested that the diversification of bacterial genomic GC content occurred independently in most bacterial phyla and classes, we decided to investigate the impact of genomic GC content on codon usage in more detail than what is described in the studies discussed above. An inspection of the bacterial genome sequences available within GenBank revealed four classes of the phylum *Proteobacteria* and four additional phyla that contained completed genome sequences from at least 15 different species. Therefore, we chose 8–12 species that represented the range of genomic GC content of each class or phylum and performed separate analyses on each group. We hypothesized that if genomic GC content was the primary determinant of codon and amino acid usage patterns, then similar usage patterns would have arisen independently in each of the eight classes or phyla. However, if codon usage patterns were the principal determinant of genomic GC content, then different codon usage patterns might have arisen in different bacterial phyla. Since similar patterns of codon usage were observed in all eight comparisons, we concluded that shared codon usage patterns have arisen independently in multiple bacterial phyla. In addition, we were able to quantify the impact of genomic GC content on predicted protein amino acid content.

## Results

### Diversification of genomic GC content

An inspection of the GC content of the completely sequenced bacterial genomes currently available in GenBank (June 2, 2010) revealed that many bacterial phyla, and also the individual classes of *Proteobacteria*, contain broad ranges of genomic GC content. Since bacterial phyla and classes are considered to be well established taxonomic groups, the diversification of genomic GC content must have occurred after the evolution of bacterial phlya and classes. However, since the bacterial species in the same genus usually have a similar genomic GC content, genomic GC content diversification must have occurred prior to, or as part of, the process of the evolution of bacterial genera.

**Figure 1:**
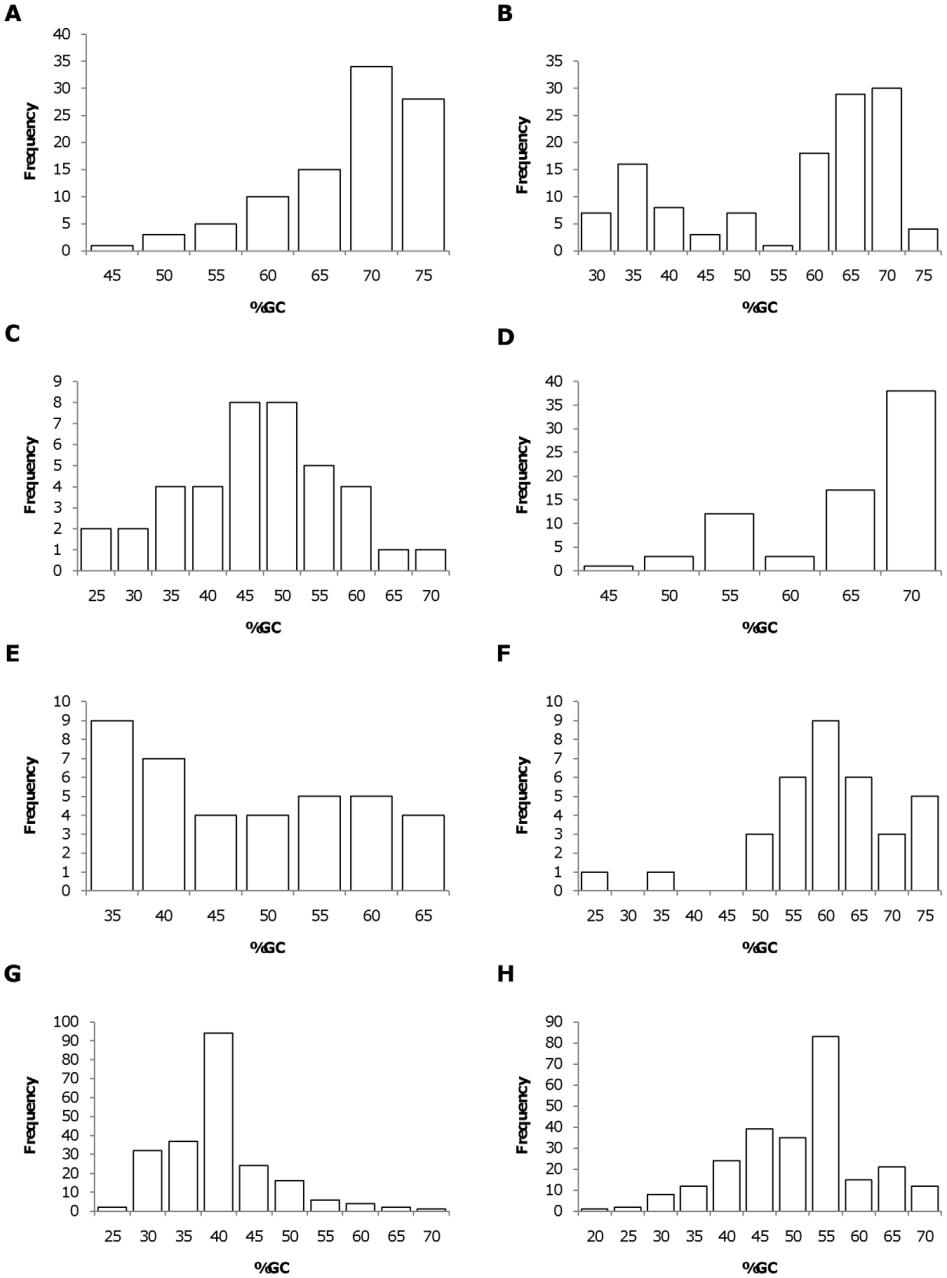
Distribution of genomic GC content within bacterial phyla or classes. Within each class or phylum, the genomic GC contents from all individual genomes available on June 16, 2010 were binned in five percent increments with the number on the X-axis representing the top range of the bin. A) *Actinobacteria*; B) *Alphaproteobacteria*; C) *Betaproteobacteria*; D) *Bacterioides/Chlorbi*; E) *Cyanobacteria*; F) *Deltaproteobacteria*; G) *Firmicutes*; H) *Gammaproteobacteria*.

When individual phyla and classes of bacteria were examined, eight were represented by at least 15 species (Tables 1 and 2). Within each group, a broad range of genomic GC content was observed (Fig. 1), indicating that a wide range genomic GC content has evolved independently in each of these eight taxonomic groups. The distribution of genomic GC content appears to differ among these phyla and classes. However, the differing patterns of GC content presented in the bar graphs may not reflect the true distribution within each group since the available sequenced genomes do not reflect a random sampling of the members of the group. Nevertheless, we were able to take advantage of the range of genomic GC content to choose a collection of 8 to 12 individual species that represent the range of diversity of genomic GC content observed within each group. In contrast to the widely quoted range of 25 to 75% for bacterial genomic GC content, the current list of completed bacterial genome sequences includes 12 bacterial species in three different phyla with GC contents that range from 16.6% to 24% (Fig. 1). Thus, the range of genomic GC content (16.6% to 74.9%) is broader than previously realized, with GC base pairs comprising only one sixth of the genome at the lower extreme. Clearly, a reduction in the genomic GC content of this magnitude would require a severe reduction in the use of amino acids such as proline, alanine, and glycine that are encoded by GC-rich codons.

**Table 1:** Bacterial species included within each phylum analyzed in this study.

Phylum	Species	GC%
*Actinobacteria*	*Gardnerella vaginalis* 409-05	42
	*Atopobium parvulum* DSM 20469	45.7
	*Cryptobacterium curtum* DSM 15641	50.9
	*Corynebacterium diphtheriae* NCTC 13129	53.5
	*Renibacterium salmoninarum* ATCC 33209	56.3
	*Propionibacterium acnes* KPA171202	60
	*Corynebacterium urealyticum* DSM 7109	64.2
	*Arthrobacter chlorophenolicus* A6	66
	*Frankia sp.* EAN1pec	71.2
	*Kineococcus radiotolerans* SRS30216	74.2
*Bacteroidetes/Chlorobi*	*Candidatus Sulcia* muelleri GWSS	22.4
	*Blattabacterium sp.* (*Blattella germanica*) str. Bge	27.1
	*Flavobacterium psychrophilum* JIP02/86	32.5
	*Candidatus Amoebophilus asiaticus* 5a2	35
	*Pedobacter heparinus* DSM 2366	42
	*Chloroherpeton thalassium* ATCC 35110	45
	*Prosthecochloris aestuarii* DSM 271	50.1
	*Robiginitalea biformata* HTCC2501	55.3
	*Rhodothermus marinus* DSM 4252	64.3
	*Salinibacter ruber* DSM 13855	66.1
*Cyanobacteria*	*Prochlorococcus marinus* str. MIT 9515	30.8
	*Prochlorococcus marinus* str. NATL1A	35
	*Nostoc sp.* PCC 7120	41.3
	*Acaryochloris marina* MBIC11017	47
	*Prochlorococcus marinus* str. MIT 9303	50
	*Synechococcus elongatus* PCC 7942	55.4
	*Synechococcus sp.* JA-3-3Ab	60.2
	*Gloeobacter violaceus* PCC 7421	62
*Firmicutes*	*Mycoplasma capricolum subsp. capricolum* ATCC 27343	23.8
	*Mycoplasma mobile* 163K	25
	*Mycoplasma arthritidis* 158L3-1	30.7
	*Bacillus cereus* G9842	35
	*Mycoplasma pneumoniae* M129	40
	*Lactobacillus brevis* ATCC 367	46.1
	*Paenibacillus sp.* JDR-2	50.3
	*Acidaminococcus fermentans* DSM 20731	55.8
	*Candidatus Desulforudis audaxviator* MP104C	60.8
	*Symbiobacterium thermophilum* IAM 14863	68.7

**Table 2:** Bacterial species included within the classes analyzed in this study.

Class	Species	GC%
*Alphaproteobacteria*	*Ehrlichia ruminantium* str. Gardel	27.5
	*Ehrlichia chaffeensis* str. Arkansas	30.1
	*Wolbachia* endosymbiont of *Drosophila melanogaster*	35.2
	*Neorickettsia sennetsu* str. Miyayama	41.1
	*Hirschia baltica* ATCC 49814	45.2
	*Anaplasma centrale* str. Israel	50
	*Ochrobactrum anthropi* ATCC 49188	56.1
	*Gluconobacter oxydans* 621H	60.8
	*Rhodopseudomonas palustris* BisB18	65
*Betaproteobacteria*	*Polynucleobacter necessarius* subsp. asymbioticus QLW-P1DMWA-1	44.8
	*Methylotenera mobilis* JLW8	45.5
	*Nitrosomonas eutropha* C71	48.5
	*Nitrosomonas europaea* ATCC 19718	50.7
	*Nitrosospira multiformis* ATCC 25196	53.9
	*Methylobacillus flagellatus* KT	55.7
	*Dechloromonas aromatica* RCB	59.2
	*Comamonas testosteroni* CNB-2	61.4
	*Ralstonia pickettii* 12D	63.3
	*Verminephrobacter eiseniae* EF01-2	65.2
	*Leptothrix cholodnii* SP-6	68.9
*Deltaproteobacteria*	*Lawsonia intracellularis* PHE/MN1-00	33.1
	*Desulfotalea psychrophila* LSv54	46.6
	*Bdellovibrio bacteriovorus* HD100	50.6
	*Pelobacter carbinolicus* DSM 2380	55.1
	*Geobacter bemidjiensis* Bem	60.3
	*Desulfovibrio vulgaris* str. ‘Miyazaki F’	67.1
	*Sorangium cellulosum* ‘So ce 56’	71.4
	*Anaeromyxobacter dehalogenans* 2CP-C	74.9
*Gammaproteobacteria*	*Candidatus Carsonella ruddii* PV	16.6
	*Buchnera aphidicola str.* Cc *(Cinara cedri)*	20.2
	*Buchnera aphidicola str.* Bp *(Baizongia pistaciae)*	25.3
	*Candidatus Vesicomyosocius okutanii* HA	31.6
	*Haemophilus somnus (histophilus somni)* 129PT	37.2
	*Haemophilus parasuis* SH0165	40
	*Shewanella denitrificans* OS217	45.1
	*Escherichia coli* APEC O1	50.3
	*Dickeya dadantii* Ech703	55
	*Pseudomonas fluorescens* SBW25	60.1
	*Xanthomonas campestris pv. campestris str.* 8004	65
	*Halorhodospira halophila* SL1	68

### Codon and amino acid usage

**Figure 2:**
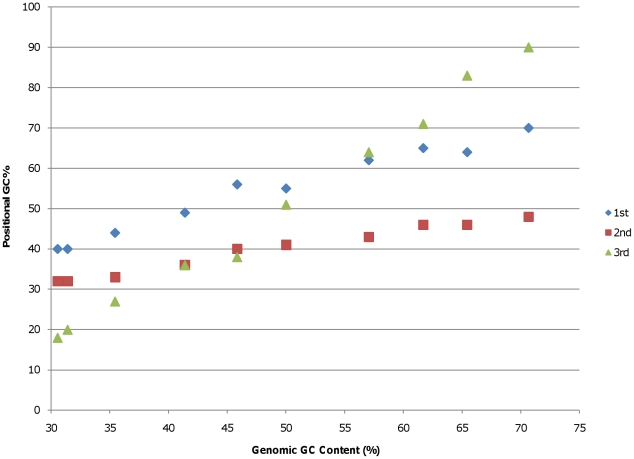
The GC content at each of the three codon positions was plotted against the genomic GC content of the representative *Alphaproteobacteria* listed in **Table 2**.

To determine how the observed variation in genomic GC content impacted codon usage, the GC content of each codon position in the coding region of the selected genomes was plotted against the genomic GC content using the *Alphaproteobacteria* as an example. Consistent with previous observations of bacterial genomes in general [5], [7], the GC content of the third codon position of the *Alphaproteobacteria* genomes increased rapidly with increasing genomic GC content (Fig. 2). The GC content of the first and second codon positions increased as well, but to a lesser extent. Thus, changes in all three codon positions contribute to the observed differences in genomic GC content in the *Alphaproteobacteria*. Similar patterns of changes in all three codon positions were observed among the representatives of each of the other seven phyla and classes included in this study (data not shown). Although the increases in third codon position GC content could be accomplished without affecting the protein amino acid sequences due to the redundancy of the genetic code, the observed increases in the GC content of the first or second codon positions would require changes in the distribution of amino acids incorporated into cellular proteins.

**Figure 3:**
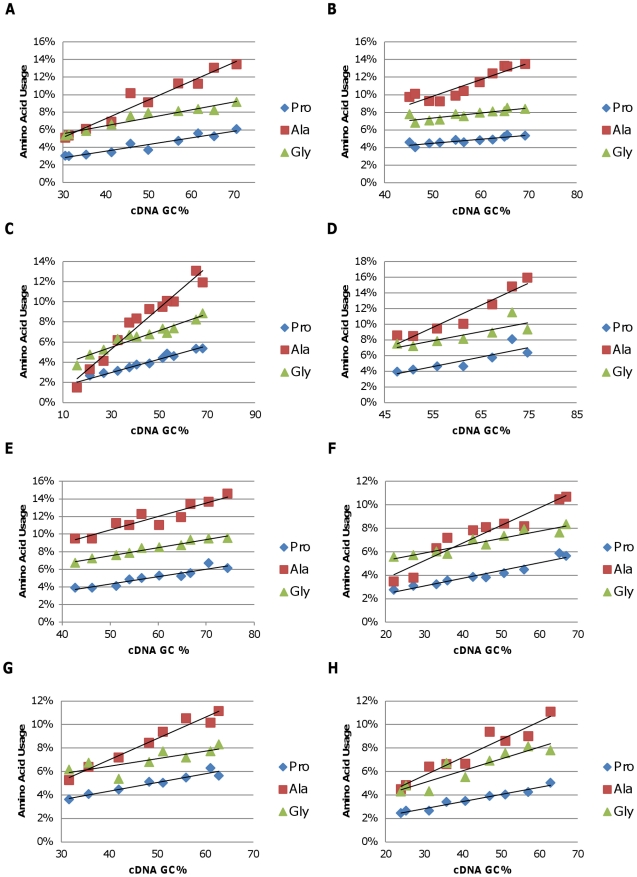
The predicted percentage of amino acids encoded by the three high-GC codon families, proline, alanine, and glycine, plotted against the genomic GC content of the representative bacteria among the following groups. A) *Alphaproteobacteria*; B) *Betaproteobacteria*; C) *Gammaproteobacteria*; D) *Deltaproteobacteria*; E) *Actinobacteria*; F) *Bacteriodetes/Chlorobi*; G) *Cyanobacteria*; H) *Firmicutes*.

**Figure 4 pone-0017677-g004:**
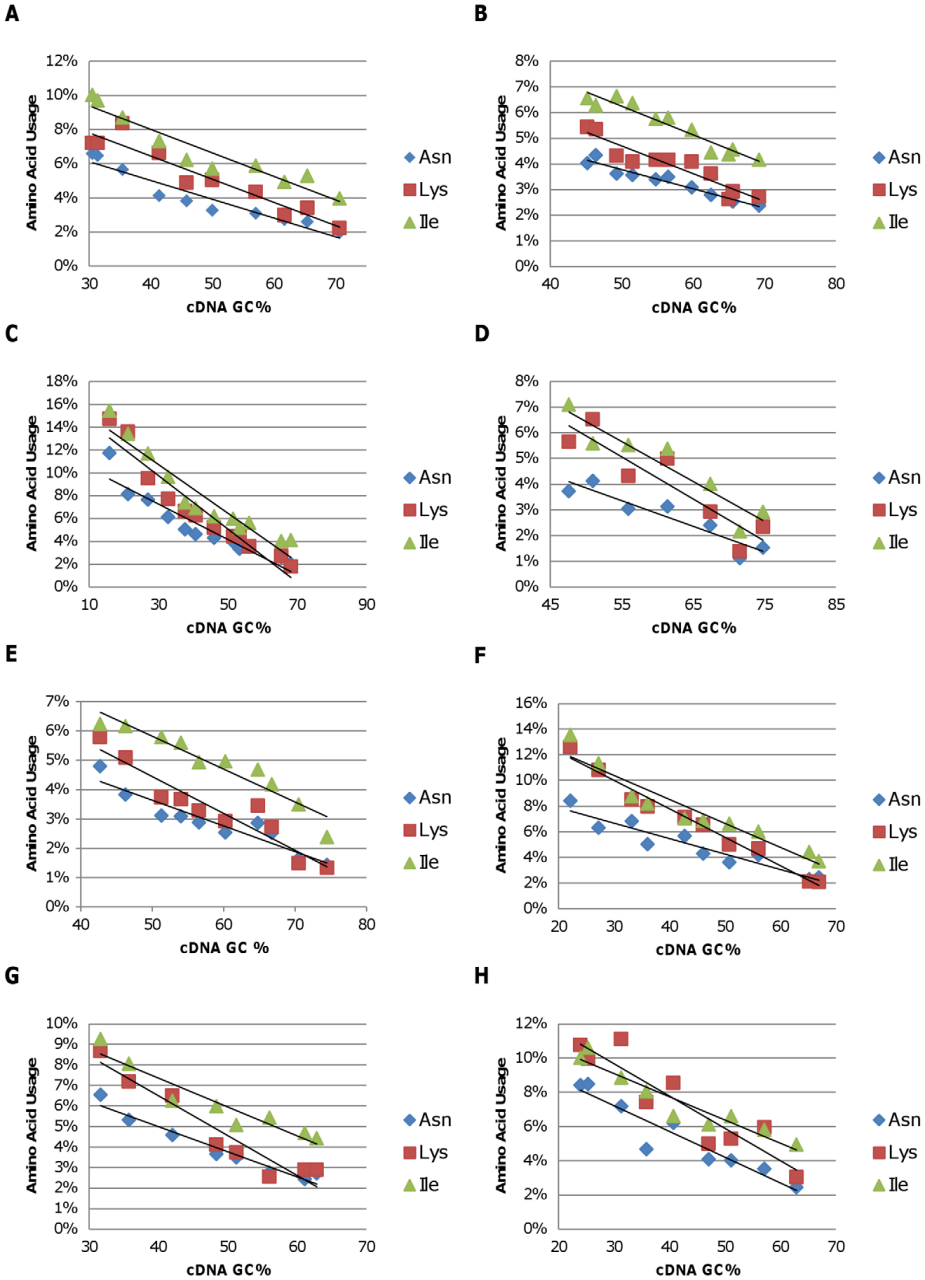
The predicted percentage of amino acids encoded three low-GC codon families, asparagine, lysine, and isoleucine, plotted against the genomic GC content of the representative bacteria among the following groups. A) *Alphaproteobacteria*; B) *Betaproteobacteria*; C) *Gammaproteobacteria*; D) *Deltaproteobacteria*; E) *Actinobacteria*; F) *Bacteriodetes/Chlorobi*; G) *Cyanobacteria*; H) *Firmicutes*.

To examine how these changes in genomic GC content impact the distribution of the amino acids used to make proteins, the proportion of amino acids that are encoded by degenerate codons with either the three high (G or C at each of the first two positions) (Fig. 3) or three of the five low (A or T at each of the first two positions) GC-content codon families (Fig. 4) was plotted against the genomic GC content of the representative bacteria in the phyla and classes examined. Clear trends were observed. Use of amino acids encoded by the high GC codons increased with increasing genomic GC content while use of amino acids encoded by the low GC codons decreased. For example, the frequency of codons coding for alanine in the *Alphaproteobacteria* increased more than two-fold with increasing genomic GC content (Fig. 3A) while the frequency of codons coding for isoleucine dropped more than two-fold (Fig. 3A). To obtain a quantitative measure of the change in amino acid codon use, we calculated the slope of the best fit line for each of the amino acids shown in Fig. 3 and 4. The slope for alanine was 0.0021 in the *Alphaproteobacteria*. Thus, if one *Alphaproteobacteria* genome had a 10% higher GC content than another, the percentage of the total codons that coded for alanine would increase approximately 2.1% (for example, from 6% to 8.1% of the total). Conversely, the best fit line for isoleucine codons had a slope of −0.0014, and a 10% increase in GC content would result in a 1.4% decrease in the number of isoleucine codons. Results similar to those obtained with the *Alphaproteobacteria* were obtained with each of the other bacterial phyla and classes (Fig. 3). In fact, for the Gammaproteobacteria, only 1.5% of the total codons were alanine codons in the *Candidatus Carsonella ruddii* PV genome (16.6% GC), while alanine codons were 11.9% of the total in the *Halorhodospira halophila* SL1 genome (68% GC).

**Figure 5 pone-0017677-g005:**
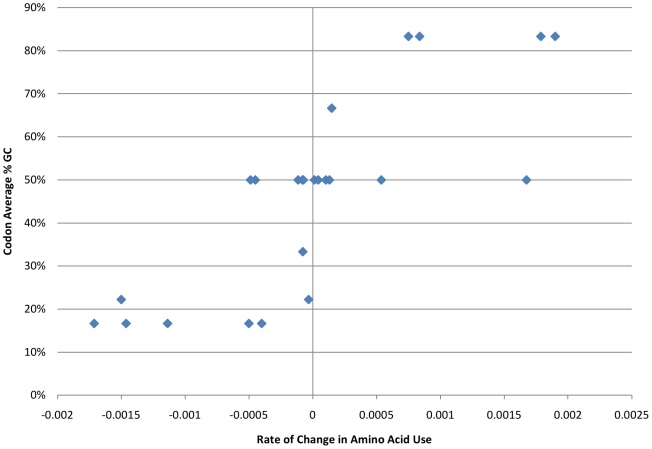
Average of the slopes for all 20 amino acid codon families (with the six-fold degenerate amino acid codon families being divided into two groups based on the first position nucleotide) for all eight groups plotted against the average GC content of their respective codons. For each class or phylum, the frequency of each amino acid in the coding portion of each genome was plotted against the genomic GC content of the genome. The slopes of these eight plots were then averaged and plotted versus the average GC content of the codon family for each amino acid.

**Figure 6 pone-0017677-g006:**
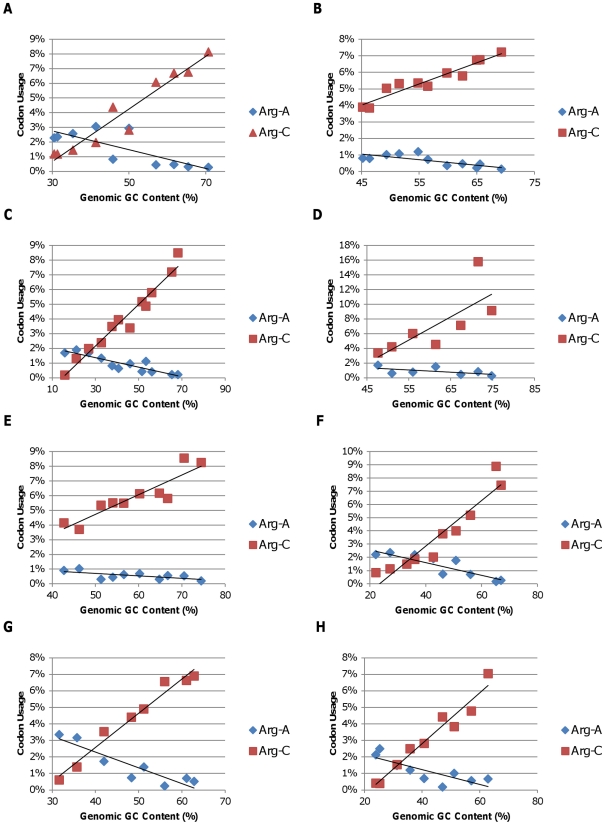
Arginine codon use plotted against the genomic GC content of the representative bacteria among the following groups. A) *Alphaproteobacteria*; B) *Betaproteobacteria*; C) *Gammaproteobacteria*; D) *Deltaproteobacteria*; E) *Actinobacteria*; F) *Bacteriodetes/Chlorobi*; G) *Cyanobacteria*; H) *Firmicutes*. Arg-A and Arg-C refer to arginine codon families with either an A or a C in the first codon position, respectively.

**Figure 7:**
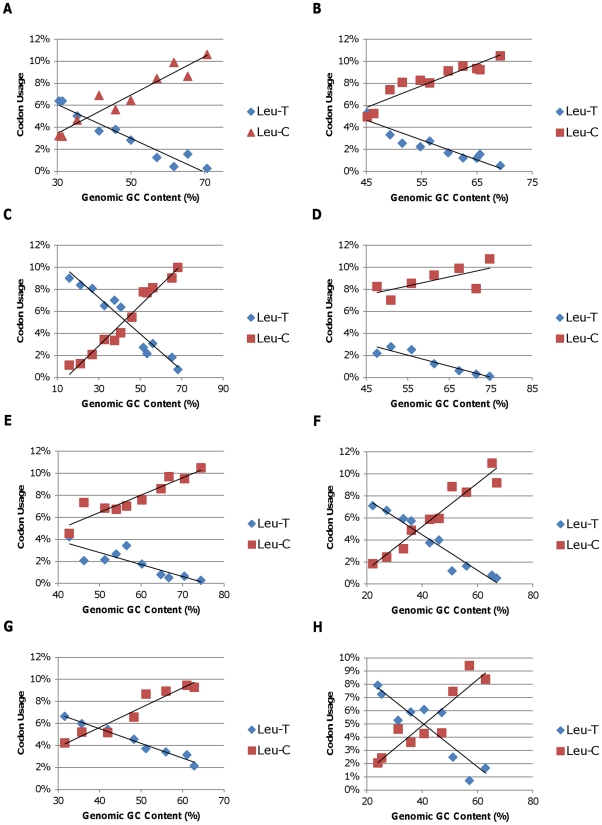
Leucine codon use plotted against the genomic GC content of the representative bacteria among the following groups. A) *Alphaproteobacteria*; B) *Betaproteobacteria*; C) *Gammaproteobacteria*; D) *Deltaproteobacteria*; E) *Actinobacteria*; F) *Bacteriodetes/Chlorobi*; G) *Cyanobacteria*; H) *Firmicutes*. Leu-T and Leu-C refer to leucine codon families with either a T or a C in the first codon position, respectively.

To summarize the impact of GC content on amino acid codon use, we plotted the frequency of each amino acid codon family in the coding region of the genomes of the representatives of each bacterial phylum or class analyzed in this study versus the genomic GC content for each species. The six-fold degenerate codon families were sub-divided into two codon families based on the first two codon positions since the use of these sub-divided families differed with genomic GC content (see below). We then calculated the slopes of the best fit lines for each amino acid plot (Tables S1–S3). These slopes were then averaged for each amino acid codon family across all eight phyla or classes of bacteria investigated in this study and plotted versus the average GC content of each codon family (Fig. 5). The results showed that the high GC amino acid codon families (upper right corner) that code for glycine, proline, and alanine had average slopes that ranged from 0.0008 to 0.0019 indicating an increase of 0.8 to 1.9% for each 10% increase in GC content for all eight phyla or classes of bacteria examined. In contrast, the low GC amino acid codon families (lower left corner) that code for asparagine, lysine, and isoleucine had average slopes that ranged from −0.0011 to −0.0017 indicating a decrease of 1.1 to 1.7% for each 10% increase in GC content. In both of these data sets, standard deviations were less than 20% of the average value. The low GC amino acid codon families that code for phenylalanine and tyrosine showed a similar, but smaller effect with average slopes of −0.0005 and −0.0004, respectively. However, these two amino acids generally occur in proteins at lower frequencies. As expected, the slopes for most codon families with 50% average GC content ranged from −0.0002 and 0.0003 indicating little or no change in the use of these amino acids with increasing genomic GC content. In contrast, the slope for the leucine-C subgroup (leucine codons with a C in the first position) was 0.0017 even though the average GC content of this set of leucine codons is also 0.5. This anomaly is easily explained when the slope of the alternative subgroup of leucine codons (leucine T) is considered (−0.0015). Even though the average GC content of the leucine-C codons is moderate, the average GC content of the alternative leucine-T codons is much lower (0.1667). Thus, genomes with a low GC content preferentially use codons that begin with T, and genomes with a high GC content preferentially use leucine codons that start with C (Fig. 6). A similar result was obtained when the use of the six arginine codons was examined. The arginine-A codon family exhibited an average slope of −0.00045, while the slope of the arginine-C codon family was 0.0018 (Fig. 7). In both of these six-codon families, the synonymous first position substitutions behave like synonymous third position substitutions. Thus, there is a consistent trend in all eight phyla and classes that GC-rich codons are used preferentially in all high GC genomes, resulting in both synonymous and nonsynonymous changes relative to genomes with lower GC content. Similarly, AT-rich codons are used preferentially in low GC genomes.

## Discussion

It is well known that the genomic GC content of bacteria varies widely and that divergent genomic GC content is correlated with altered codon and amino acid usage. In contrast to previous studies, our analysis of individual bacterial classes and phyla was designed to determine whether similar patterns of codon and amino acid usage variation with respect to genomic GC content have evolved independently in each of the eight groups. The divergence of genomic GC content within disparate Gram negative and Gram positive bacterial phyla indicate that GC content divergence was not produced by a single event that resulted in individual phyla or classes filling a certain GC content niche. Instead, each of the eight phyla and classes in this study contained species with widely disparate genomic GC concentrations (Fig. 1). Since the current classification of bacteria into phyla and classes is generally consistent with the available nucleotide sequence information, the observed distribution of genomic GC content must have evolved independently in each of the phyla and classes analyzed in this study. In addition, since closely related bacterial species usually have very similar genomic GC content, the observed divergence in genomic GC content must have occurred prior to the divergence of these contemporary species. The presence of closely-related species with similar genomic GC content also implies that current genomic GC content is relatively stable on an evolutionary time scale. Further, since random mutation would eventually lead to a 50% GC content, this stability must reflect the presence of some mechanism(s) for preserving GC content. Taken together, these data indicate that divergent bacterial genomic GC content has evolved repeatedly and is actively maintained by contemporary bacterial species.

The observed variation in genomic GC content has a strong impact on synonymous codon usage with most of the variation occurring in the third codon position due to the redundancy of the genetic code [5], [6], [7]. However, reproducible changes in the GC content of the first and second codon positions occur as well, as illustrated in Fig. 2. Since more than 1000 complete bacterial genomes are now available, we decided to explore this phenomenon further to determine if the patterns of codon usage previously observed in bacteria with GC-rich genomes [12] were replicated across a broad range of bacterial taxa. Our analyses show that, within each bacterial phylum and class analyzed in this study, the use of GC-rich codons increases as genomic GC content increases (Fig. 5). Furthermore, the data indicate that for leucine and arginine, each of which are encoded by two codon families that differ in GC content, the codons with the higher GC content are found preferentially in genomes with high GC content (Fig. 6 and 7). For leucine, the synonymous codons range from no GC content (UUA) to two thirds GC (CUC, CUG) allowing for a greater shift in GC content than is possible with four-fold degenerate codons. Similarly, arginine codons range from one third GC (AGA) to 100% GC (CGG and CGC). In fact, these first position synonymous substitutions account for most of the difference in the slopes of plots of the first position GC content versus total genomic GC content (Fig. 6 and 7). Again, this consistency across the eight classes and phyla of Gram-negative and Gram-positive bacteria included in this study indicates that the observed patterns are a result of shared GC content rather than shared ancestry.

As shown previously [8], we demonstrated that for each class and phylum analyzed, the proportion of amino acids that are encoded by high GC codons increases as genomic GC content increases (Fig. 4). Thus, increased use of alanine, arginine, glycine, and proline is paired with decreased use of asparagine, lysine, and isoleucine. Furthermore, our analyses show that this pattern is consistent across all eight phyla and classes of bacteria analyzed in this study (Fig. 3–7). Thus, in addition to causing changes in synonymous codon use, changes in genomic GC content have a strong effect on the amino acid composition of the encoded proteins. These results indicate that considerable variation in amino acid content can be tolerated in most bacterial proteins without causing a significant impact on protein function. This consistency of the impact of genomic GC content on patterns of protein amino composition across bacterial phyla and classes provides additional support for the idea that genomic GC content is a driving force in genome evolution.

GC content not only affects the proportions of amino acids encoded by a genome, but also the sequence complexity of the encoded proteins. Of the 10 two-fold and three-fold degenerate amino acid codon families, five have an A or T at both of the first two positions and five are GC neutral at the first two positions. In the four-fold degenerate codon families, three have either G or C at both of the first two positions and two are GC neutral. Since genomes with high GC content have increased GC content in the first and second codon positions, there is increased use of the three GC-rich codon families and decreased use of the five GC-poor codon families in protein coding regions. The reverse is true in low GC genomes. Thus, both high GC and low GC genomes have a limited amino acid vocabulary compared to that of mid-GC genomes. Furthermore, the lower number of GC-rich codon families causes this effect to be greater in high-GC genomes, resulting in greater genome homogeneity as previously observed by Bohlin and Skjerve [14].

Bohlin and Skjerve's work [14] also showed a correlation between GC content and aerobiosis which supports the hypothesis that increased genomic GC concentration evolved in response to rates of mutation associated with the use of oxygen in metabolism. Previously, Naya et al. [15] proposed that increased GC content would be advantageous in aerobic organisms because those amino acids that are preferentially oxidized (Cys, Met, Trp, Tyr, Phe, and His) have reduced frequencies in aerobic bacteria. The codons for four of these amino acids (Cys, Met, Tyr, and Phe) have an A or T in both of the first two positions, and reduction in their use would increase genomic GC content. Naya et al. [15] also noted that increased GC content makes the genome less susceptible to aerobiosis-related deleterious mutations either by guanine scavenging of oxidizing agents that protects other bases, or by withstanding mutations more easily in the third position because of the increased use of 4-fold degenerate amino acids. However, if aerobiosis was the driving force in the evolution of high-GC genomes, we would not expect to find anaerobic bacteria with high-GC genomes. Nevertheless, anaerobes such as *Desulfovibrio vulgaris, Halorhodospira halophila*,and *Anaeromyxobacter dehalogenans* have extremely high GC genomes and their patterns of codon usage are identical to those of the aerobic bacteria whose genomes have similar GC content. There are also examples of low GC aerobic bacteria such as *Ehrlichia chaffeensis*, *Buchnera aphidicola,* and *Candidatus carsonella rudii*. These bacteria, however, are intracellular parasites and, in the cases of *B. aphidicola and C. carsonella rudii,* have extremely small genomes. Since small genomes are correlated with low GC content [16], it is not clear which factor might be a driving force. While these exceptions do not rule out the possibility that aerobiosis may be a factor that influences bacterial genomic GC content, they suggest that it is not the only factor.

Genomic GC content also has been correlated with optimal growth temperature. Musto et al. [17], [18] used an approach similar to ours and showed that within families a significant correlation between growth temperature and GC content was observed in 9 out of 20 families. Thus, optimal growth temperature may influence genomic GC content in some bacterial families. Genome size also correlated with genomic GC content except with anaerobic bacteria [16]. Thus, multiple factors have been correlated with genomic GC content for various subsets of bacteria. However, no cause and effect relationships have been established so it not clear whether genomic GC content influences the probability of the success of a particular lifestyle, or whether a particular lifestyle influences genomic GC content. Since life itself is complex, and most bacteria are constantly subjected to changing environments, it is likely that the apparent status quo is maintained by the interplay of conflicting forces.

Despite the uncertainty about the factors responsible for the origin and maintenance of the observed disparate bacterial genome GC content, it seems clear that genomic GC content determines bacterial codon usage since similar patterns of codon use were observed in each of the eight phyla and classes included in this study. Specifically, in every group, codons with higher GC content were used with increasing frequency as genomic GC content increased (Fig. 5). In addition, Chen et al. [13] showed that codon usage patterns could be predicted from an analysis of the noncoding regions of the genome. In the alternative scenario where amino acid content is the driving force, changes in codon usage would have caused changes in genomic GC content, and therefore, selection for changes in GC content of the noncoding regions would not be expected. Furthermore, if individual genera were evolving the preferential use of specific codons for specific amino acids, we might expect to find genomes that use GC-rich codons for some amino acids and AT-rich codons for others. This pattern was not observed for any of 78 species studied. Instead, the codons preferred for individual amino acids always reflect the genomic GC content suggesting that in bacteria, codon usage has had to adapt to a particular genomic GC content. Thus, genomic GC content appears to be the primary determinant of both codon and amino acid usage patterns. As a result, the observed selection for translational efficiency of highly expressed genes within a given genome must be constrained by the established parameters of that genome as suggested by the analyses of Chen et al. [13].

## Materials and Methods

Five bacteria phyla (*Actinobacteria, Bacteriodetes/Chlorobi, Cyanobacteria, Firmicutes,* and *Proteobacteria*) with a sufficiently large number of sequenced genomes (>15 species) were identified in the NCBI Genome Database (http://www.ncbi.nlm.nih.gov/sites/entrez?db=genome). In addition, genome sequences for more than 15 species were available in four classes of the *Proteobacteria*, *alpha, beta, gamma*, and *delta.* To choose species for further analyses, the available species in each of these phyla and classes were ordered according genomic GC content, and the species with the lowest genomic GC content was selected from each 5% GC interval except in the last interval where two species, one with the highest and one with the lowest GC% content within the interval, were selected. The one exception was the *Betaproteobacteria* where two species were selected from each 5% interval because of the narrower range of GC% content (45–63%) in the available genomes. A list of the chosen bacterial species and their genomic GC content is shown in Tables 1 and 2.

The coding regions of each of the 78 genomes were obtained from GenBank (ftp://ftp.ncbi.nih.gov) on June 16, 2010. Codon usage for each coding sequence was calculated using an original program (available upon request) written in Strawberry Perl (http://strawberryperl.com/), and the resulting codon usage tables were used to determine the amino acid composition of the translated genome. Artemis [19] was used to compare the GC content of each reading frame within a species.

## Supporting Information

Table S1 **Slope of a plot of codon use versus genomic GC content for codon families with non-neutral average GC content.**
(DOC)

Table S2 **Slope of a plot of codon use versus genomic GC content for codon families with neutral average GC content.**
(DOC)

Table S3 **Slope of a plot of codon use versus genomic GC content for six member codon families sub-divided by the first position base.**
(DOC)
