# Supplementary material for: Across Bacterial Phyla, Distantly-Related Genomes with Similar Genomic GC Content Have Similar Patterns of Amino Acid Usage
Source: PLoS One. 2011 Mar 10;6(3):e17677. doi: 10.1371/journal.pone.0017677 (PMC3053387; doi:10.1371/journal.pone.0017677)
Supplement: Table S1 — Slope of a plot of codon use versus genomic GC content for codon families with non-neutral average GC content. (DOC) [file pone.0017677.s001.doc]

Supplementary Table S1. Slope of a plot of codon use versus genomic GC content for codon families with non-neutral average GC content.

|  | Pro | Ala | Gly | Ile | Asn | Lys | Met | Phe | Tyr | Trp |
| --- | --- | --- | --- | --- | --- | --- | --- | --- | --- | --- |
| Codon GC% | 0.83 | 0.83 | 0.83 | 0.22 | 0.17 | 0.17 | 0.33 | 0.167 | 0.17 | 0.67 |
|  |  |  |  |  |  |  |  |  |  |  |
| Actinomycetes | 0.0008 | 0.0015 | 0.0009 | -0.0011 | -0.0009 | -0.0013 | -0.0004 | -0.0004 | -0.0004 | 0.0001 |
| Alphaproteobacteria | 0.0008 | 0.0021 | 0.0009 | -0.0014 | -0.0011 | -0.0014 | -0.000002 | -0.0003 | -0.0005 | 0.0002 |
| Bacteroidetes | 0.0007 | 0.0015 | 0.0006 | -0.0019 | -0.0012 | -0.0022 | 0.000002 | -0.0004 | -0.0004 | 0.0002 |
| Betaproteobacteria | 0.0005 | 0.0019 | 0.0006 | -0.0011 | -0.0007 | -0.0011 | -0.00004 | -0.0003 | -0.0004 | 0.0001 |
| Cyanobacteria | 0.0007 | 0.0018 | 0.0006 | -0.0014 | -0.0012 | -0.0019 | -0.00008 | -0.0004 | -0.0001 | 0.0001 |
| Deltaproteobacteria | 0.0012 | 0.0028 | 0.0012 | -0.0016 | -0.001 | -0.0016 | -0.0004 | -0.0008 | -0.0006 | -0.00001 |
| Firmicutes | 0.0006 | 0.0015 | 0.0011 | -0.0014 | -0.0015 | -0.0019 | 0.0002 | -0.0006 | -0.0003 | 0.0003 |
| Gammaproteobacteria | 0.0007 | 0.0021 | 0.0008 | -0.0021 | -0.0015 | -0.0023 | 0.0001 | -0.0008 | -0.0005 | 0.0002 |
| Average | 0.00075 | 0.0019 | 0.00084 | -0.0015 | -0.0011 | -0.0017 | -0.00008 | -0.0005 | -0.0004 | 0.00015 |
