# Supplementary material for: Across Bacterial Phyla, Distantly-Related Genomes with Similar Genomic GC Content Have Similar Patterns of Amino Acid Usage
Source: PLoS One. 2011 Mar 10;6(3):e17677. doi: 10.1371/journal.pone.0017677 (PMC3053387; doi:10.1371/journal.pone.0017677)
Supplement: Table S2 — Slope of a plot of codon use versus genomic GC content for codon families with neutral average GC content. (DOC) [file pone.0017677.s002.doc]

Supplementary Table S2. Slope of a plot of codon use versus genomic GC content for codon families with neutral average GC content.

|  | Thr | Val | His | Gln | Asp | Glu | Cys |
| --- | --- | --- | --- | --- | --- | --- | --- |
| Codon GC% | 0.5 | 0.5 | 0.5 | 0.5 | 0.5 | 0.5 | 0.5 |
|  |  |  |  |  |  |  |  |
| Actinomycetes | 0.0001 | 0.0006 | 0.00005 | -0.0003 | 0.000001 | -0.0003 | -0.0002 |
| Alphaproteobacteria | 0.0002 | 0.0002 | -0.00002 | 0.000005 | 0.0001 | -0.0001 | -0.0002 |
| Bacteroidetes | 0.0003 | 0.0008 | 0.00009 | 0.0002 | 0.0005 | 0.0003 | -0.00008 |
| Betaproteobacteria | -0.00009 | 0.0004 | 0.00003 | -0.00003 | 0.00004 | -0.0004 | 0.00003 |
| Cyanobacteria | -0.00006 | 0.0006 | 0.00007 | 0.0006 | -0.0001 | -0.0002 | -0.00004 |
| Deltaproteobacteria | -0.0006 | 0.0002 | 0.0002 | -0.0005 | 0.00006 | -0.0003 | -0.0001 |
| Firmicutes | -0.00004 | 0.0008 | 0.0002 | -0.00003 | -0.00005 | -0.00004 | 0.0001 |
| Gammaproteobacteria | 0.0003 | 0.0007 | 0.0002 | 0.0004 | 0.0005 | 0.0004 | -0.0001 |
| Average | 0.0003 | 0.0007 | 0.0001 | 0.00004 | 0.00018 | -0.00008 | -0.00005 |
