# Supplementary material for: Across Bacterial Phyla, Distantly-Related Genomes with Similar Genomic GC Content Have Similar Patterns of Amino Acid Usage
Source: PLoS One. 2011 Mar 10;6(3):e17677. doi: 10.1371/journal.pone.0017677 (PMC3053387; doi:10.1371/journal.pone.0017677)
Supplement: Table S3 — Slope of a plot of codon use versus genomic GC content for six member codon families sub-divided by the first position base. (DOC) [file pone.0017677.s003.doc]

Supplementary Table S3. Slope of a plot of codon use versus genomic GC content for six member codon families sub-divided by the first position base.

|  | Arg-A | Arg-C | Leu-T | Leu-C | Ser-T | Ser-A |
| --- | --- | --- | --- | --- | --- | --- |
| Codon GC% | 0.5 | 0.83 | 0.167 | 0.5 | 0.5 | 0.5 |
|  |  |  |  |  |  |  |
| Actinomycetes | -0.0002 | 0.0013 | -0.0011 | 0.0016 | -0.0005 | -0.0002 |
| Alphaproteobacteria | -0.0006 | 0.0018 | -0.0015 | 0.0017 | -0.0005 | -0.0003 |
| Bacteroidetes | -0.0005 | 0.0017 | -0.0016 | 0.002 | -0.0004 | 0.00002 |
| Betaproteobacteria | -0.0003 | 0.0013 | -0.0018 | 0.002 | -0.0004 | -0.00004 |
| Cyanobacteria | -0.001 | 0.0021 | -0.0013 | 0.0018 | -0.0008 | 0.000005 |
| Deltaproteobacteria | -0.0003 | 0.0032 | -0.001 | 0.0008 | -0.0005 | -0.0005 |
| Firmicutes | -0.0004 | 0.0015 | -0.0017 | 0.0017 | -0.0003 | -0.0001 |
| Gammaproteobacteria | -0.0003 | 0.0014 | -0.0017 | 0.0018 | -0.0005 | 0.0002 |
| Average | -0.00045 | 0.0018 | -0.0015 | 0.0017 | -0.0005 | -0.0001 |
